# Prize Essay

**Published:** 1845-07

**Authors:** 


### PRIZE ESSAY.

A premium of $50 is offered by the Medical Society of Tennessee for the best essay on *Scrofula*, under the following regulations:

"The essays must be transmitted to Dr. Felix Robertson, Nashville, Tennessee, (post-paid) on or before the first Monday in March, 1846.

"Each essay must be accompanied with a sealed packet, on which shall be written some device or sentence, and within shall be endorsed the author's name and place of residence. The same device or sentence shall be written on the dissertation to which it is attached.

"All unsuccessful essays are to be deposited with the Corresponding Secretary, from whom they may be obtained, if called for within one year after they have been received.

"The President appointed the following members a committee to award the prize—a majority of whom were empowered to act, viz: Drs. Felix Robertson, John Waters, T. R. Jennings, John R. Wilson, and A. H. Buchanan."

We hope this offer will bring out some good papers on this highly interesting subject. Scrofula, in the form of Cachexia Africana, is the most destructive disease to which one portion of our population, the colored people, are obnoxious, not unfrequently cutting down whole families. We have known the parents, and all the children to die of it in some instances, within a few years; and in one family, in Tennessee, seventeen negroes all closely related, died of this complaint in a single year. It is held by physicians to be one of the most intractable of diseases. The Medical Society of Tennessee will deserve well of the world, if it shall elicit light concerning the nature and cure of such a malady. Y.

